# Color, lipid, and protein oxidation of chicken meat from differently intensified production systems during accelerated oxidation display conditions

**DOI:** 10.1016/j.psj.2026.107427

**Published:** 2026-07-14

**Authors:** Zeshan Ali, Massimiliano Petracci, Cécile Berri, Els Vossen, Eline Kowalski, Stefaan De Smet

**Affiliations:** a Laboratory for Animal Nutrition and Animal Product Quality, Department of Animal Sciences and Aquatic Ecology, Ghent University, Ghent, 9000, Belgium; b Department of Agricultural and Food Sciences, University of Bologna, Cesena, 47522, Italy; c INRAE, Université de Tours, BOA, Nouzilly, 37380, France

**Keywords:** Broilers, Meat quality, Oxidative stability, Antioxidant enzymes, Oxidative stress

## Abstract

This study aimed to compare the oxidative stability of chicken breast and thigh meat from four distinct production systems differing in their level of intensification using an accelerated oxidation display model. The systems differed in housing conditions and growth rate of the genotypes: indoor and fast-growing (Ind_Fast), indoor and fast-medium-growing (Ind_Fastmed), outdoor and medium-growing (Out_Med), and outdoor and slow-growing (Out_Slow). Sixty mixed-sex broilers were randomly selected from 20 farms in Belgium (5 farms per production system × 3 chickens per farm). On day 2 post-slaughter, skinless breast and thigh meat from each chicken were ground separately. Forty g of ground sample was stored under light (1,600–2,200 lux, 2–4°C) for 168 h, while the remaining ground meat was used for composition analyses. Color CIE L* a* b* coordinates were measured at 0, 48, 96 and 168 h, with total color change (Δ*E*) calculated at the end of the display period. Lipid oxidation (thiobarbituric acid reactive substances, TBARS), and protein oxidation (protein carbonyl compounds, PCC) were measured at the end of display. In both breast and thigh, indoor-raised chickens exhibited lighter color, characterized by higher L*, and lower a* and b* (P < 0.001) compared to outdoor-raised chickens. However, outdoor-raised chickens showed greater Δ*E* compared to indoor counterparts, indicating lower color stability. TBARS were higher in breast meat from Out_Slow than Ind_Fast chickens (P < 0.05), whereas there were no significant differences between production systems in thigh meat. The TBARS values remained below the 1 mg/kg threshold for off-flavors in both meat cuts. PCC was not affected by production system in either meat cut. These differences in oxidative stability may be due to production system-related variation in fatty acid composition, heme iron concentration, and antioxidant status, with effects depending on the meat cut. The findings suggest that the observed variation in oxidative stability reflects the combined influence of multiple production system characteristics rather than the effect of any single factor.

## Introduction

The global demand for chicken meat continues to increase due to its affordable price, convenience in preparation, perceived health benefits, and widespread cultural and religious acceptance (Dewi et al., 2024). To meet the demand, the poultry industry mainly relies on intensive farming practices, including indoor housing at high stocking densities, the use of fast-growing genotypes, and feeding strategies designed to maximize productivity (da Silva et al., 2017). However, there are increasing consumer concerns about the impact of animal farming practices on animal welfare and environmental impact, particularly in Western Europe (ECC, 2018; Çapan and Bağdatli, 2021). This shift has spurred demand for poultry products to rely on less intensive farming practices and focus on animal welfare, leading to the increased adoption of slower-growing chicken genotypes, reduced stocking densities, and alternative housing and production methods such as free-range and organic farming (ECC, 2018).

Meat quality is a broad concept composed of many traits. The focus of the present study is on the oxidative stability of meat, determined by color fading and the oxidation of lipids and proteins. Production system factors such as genotype, age, growth rate, sex, diet, stocking density, and housing conditions significantly affect chicken meat quality (Baéza et al., 2022). These factors, particularly growth rate, influence muscle metabolism and behavior in chickens (Dixon, 2020). For example, intensive farming, involving fast-growing genotypes and high stocking densities, can induce oxidative stress *in vivo*, leading to impaired meat quality development post-mortem and reduced resistance to oxidation during storage (Nasr et al., 2021). Contrary to this, it is often assumed that chickens raised in organic systems benefit from foraging behavior through enhancing the deposition of antioxidant compounds in muscle tissue. However, it has been suggested that increased physical activity during foraging may trigger oxidative stress, which can negatively affect meat quality (Castellini and Dal Bosco, 2017).

Production system factors also impact meat composition, including the fatty acid profile and heme iron (**heme-Fe**) content, both of which are related to meat oxidative stability. Research has shown that chickens raised in organic systems generally have higher levels of polyunsaturated fatty acids (**PUFA**), a lower n-6:n-3 PUFA ratio, and lower levels of saturated fatty acids in both breast and thigh meat cuts (Dal Bosco et al., 2016; Michalczuk et al., 2017). Heme-Fe has been reported to be higher in organic meat due to the higher myoglobin content, which is essential for oxygen transport during increased physical activity (Pellattiero et al., 2020). Although higher levels of both heme-Fe and PUFA are desirable from a nutritional perspective, they may stimulate lipid oxidation and thereby compromise eating quality (Domínguez et al., 2019).

Elevated heme-Fe content in chicken meat has been linked to increased protein oxidation, as free or loosely bound iron can catalyze the formation of reactive oxygen species (**ROS**) via Fenton-type reactions, promoting oxidative damage to muscle proteins (Soyer et al., 2010). In addition, the meat myoglobin concentration and the chemical state of iron in the heme moiety are the main determinants of meat color and its stability (Droval et al., 2012). Color deterioration during meat storage and display is often associated with lipid oxidation, as reactive lipid oxidation products can promote metmyoglobin formation, leading to reduced color stability (Faustman et al., 2010). Muscle enzymes, particularly glutathione peroxidase (**GSH-Px**) and superoxide dismutase (**SOD**), are known for contributing to control pro-oxidant factors and protect lipid and proteins from oxidative damage in post-mortem muscle (Liu et al., 2014). These enzymes serve as the first line of defense against ROS (Delles et al., 2014). SOD scavenges superoxide anions, converting them into hydrogen peroxide which is then further reduced by GSH-Px into water and alcohols, thereby preventing oxidative damage to lipids and proteins. Stressful rearing conditions can impair these enzymes, leading to increased free radical production and reduced oxidative stability (Miao et al., 2020).

The aim of this study was to examine whether variations in chicken production system characteristics influence the postmortem oxidative status of breast and thigh meat under accelerated oxidation display conditions. We hypothesized that less intensive production systems may exert both protective and pro-oxidant effects on meat oxidative stability through differences in antioxidant status, heme-Fe content, and fatty acid composition, with the overall response depending on muscle type and the balance among these factors. Together, these factors may influence postmortem muscle metabolism and contribute to differences in meat color, lipid oxidation, and protein oxidation. As commercial production systems differ simultaneously in multiple characteristics, the present study should be seen as an observational comparison of systems rather than a controlled experiment to study the effect of separate production factors.

## Materials and methods

### Animals, production systems and experimental design

**Fig. 1:**
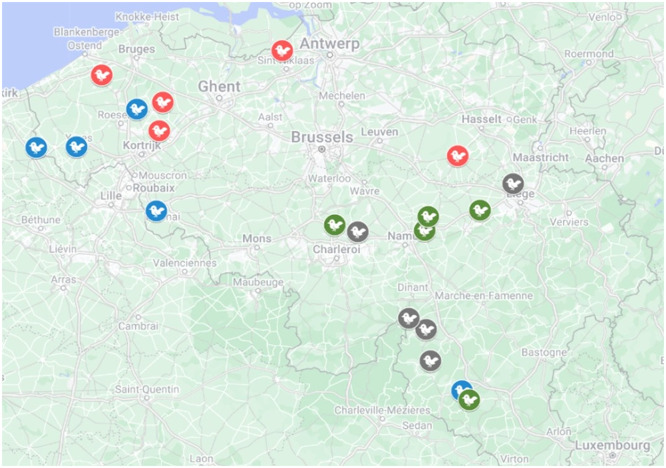
Geographic distribution of selected chicken farms across Belgium, categorized by four production systems: Ind_Fast (indoor and fast-growing, red), Ind_Fastmed (indoor and fast-medium-growing, blue), Out_Med (outdoor and medium-growing, grey), and Out_Slow (outdoor and slow-growing, green). Map generated using Google Maps (Google LLC).

In this study, four distinct chicken production systems in Belgium were chosen: indoor and fast-growing genotype (**Ind_Fast**), indoor and fast-medium-growing genotype (**Ind_Fastmed**), outdoor and medium-growing genotype (**Out_Med**), and outdoor and slow-growing genotype (**Out_Slow**). The selection criteria ensured that these systems were diverse and representative for meat which is marketed and available to consumers in Belgium. The Ind_Fast system represents the conventional intensive production system. The Ind_Fastmed system shares similar practices as Ind_Fast but provides chickens with more space and environmental enrichment, aligning with the Better Chicken Commitment (**BCC**), also known as the European Chicken Commitment (**ECC**). The Out_Med system corresponds to free-range production, and the Out_Slow system adheres to organic farming standards. Five farms were randomly selected from each production system across Belgium (Fig. 1).

The differences in production system factors are detailed in Table 1. The housing conditions for the Ind_Fast system followed EU requirements (European Commission, 2017), and those for the Ind_Fastmed system complied with the standards outlined in the Better Chicken Commitment policy (Better Chicken Commitment, 2018). Chickens in Out_Med and Out_Slow production systems were raised under traditional conditions in accordance with EU Commission Regulations 543/2008 and 889/2008, with Out_Slow additionally meeting the criteria for organic farming. However, in the Out_Slow and Out_Med production systems, outdoor access was restricted on farms due to avian influenza. This reduced the planned 6-week duration to 5, 4, 3, 2, and 1 weeks in five farms of Out_Slow, and the planned 4-week duration to 2 weeks on two farms in Out_Med. The temperature, humidity and light conditions were meticulously controlled in Ind_Fast and Ind_Fastmed system throughout the rearing period. Chickens were fed *ad libitum* in each production system; however, the nutrient and chemical composition of the diets differed among systems in each phase. Feed for Ind_Fast and Ind_Fastmed chickens was sourced from Vanden Avenne (Ooigem, Belgium), whereas feed for Out_Med and Out_Slow chickens was procured from Moulin du Val-Dieu (Aubel, Belgium) and ForFarmers (Izegem, Belgium) respectively. The nutrient composition of the finisher diets was provided by the feed manufacturers. In addition, representative feed samples were collected from each farm and analyzed for fatty acid composition. The resulting fatty acid profiles are presented in Table 1.

**Table 1:** Characteristic features of production systems and nutrient composition of finisher phase diets.

	Production system			
Characteristics	Ind_Fast	Ind_Fastmed	Out_Med	Out_Slow
Genotype	Ross 308	Redbro, RC	H JA757	S XL451
Age at slaughter (days)	42	45	56	73-76
Finishing phase (days)	29-42	35-45	36-56	36-76
Live weight at slaughter (kg)	2.6-2.8	2.4-2.6	2.1-2.3	2.3-2.5
Stocking density (kg/m^2^)	42	30	27	21
^1^Outdoor access	No	No	Yes	Yes
Enrichments	No	Yes	Yes	Yes
*Chemical Composition of the finishing diet (g/100g)*				
Protein	21.33	18.30	17.10	20.50
Ash	5.60	4.60	4.70	5.40
Fiber	2.60	3.10	3.30	2.90
^2^Fat content	9.33	7.60	5.54	6.09
Methionine	0.75	0.60	0.40	0.30
Lysine	1.25	1.20	1.00	0.94
^3^Vitamin A (IU/kg)	1,320	990	1,026	10,000
^3^Vitamin D_3_ (IU/kg)	3,630	2,722	3,065	3,000
^3^Vitamin E (IU/kg)	50	40	65	55
Phosphorus %	0.43	0.38	0.40	0.60
Na %	0.16	0.17	0.13	0.20
Se (mg/kg)	0.52	0.39	0.30	0.30
Iron (mg/kg)	19.80	14.80	45	70
Zn (mg/kg)	66.00	49.50	91	70
Cu (mg/kg)	19.80	14.80	15	15
*Fatty acids (g/100g FAME)*				
SFA	32.33	32.11	27.54	17.30
MUFA	26.48	21.41	28.84	25.75
PUFA	40.46	45.15	42.90	56.32
n-3-PUFA	3.62	4.80	3.33	6.01
*ALA*	3.46	4.58	3.14	5.73
*EPA*	0.01	0.02	0.01	0.03
*DPA*	ND	ND	ND	0.01
*DHA*	ND	ND	ND	ND
n-6-PUFA	36.82	40.35	39.65	50.32
*LA*	36.73	40.26	39.59	50.21
*AA*	0.01	0.02	0.03	0.00
PI	44.67	50.47	47.03	62.95
n-6:n-3	10.5	8.92	13.00	8.40

Abbreviations: Ind_Fast = indoor and fast-growing; Ind_Fastmed = indoor and fast-medium-growing; Out_Med = outdoor and medium-growing; Out_Slow = outdoor and slow-growing; RC = Ranger Classic; H JA757 = Hubbard JA457, S XL451 = SASSO XL451; FAME = fatty acid methyl esters; SFA = saturated fatty acid; MUFA = monounsaturated fatty acid; PUFA = polyunsaturated fatty acid (n-3-PUFA + n-6- PUFA); ALA = α-linolenic acid (C18:3n−3); EPA = eicosapentaenoic acid (C20:5n−3); DHA = docosahexaenoic acid (C22:6n−3); n-3-PUFA = omega-3 polyunsaturated fatty acid (C18:3n-3, C20:3n-3, C20:4n-3, C20:5n−3, C22:5n−3, C22:6n−3); n-6-PUFA = omega-6 polyunsaturated fatty acid (C18:2n−6, C18:3n−6, C20:2n− 6, C20:3n−6, C20:4n−6, C22:4n−6, C22:5n−6); LA = linoleic acid (C18:2n−6); AA = arachidonic acid (C20:4n-6); PI = peroxidizability index; ND = not detected.

^1^Outdoor access was either interrupted or temporarily not possible due to avian flu.

^2^Estimated from fatty acid profile.

³Vitamins A, D₃ and E were supplied as retinyl acetate, cholecalciferol and dl-α-tocopheryl acetate, respectively.

A total of 60 mixed-sex chickens (n = 3 per farm; n = 15 per system) were randomly selected for this experiment, irrespective of sex or body weight. Chickens from the Ind_Fast and Ind_Fastmed systems were slaughtered at Plukon (Maasmechelen, Belgium), whereas those from the Out_Med and Out_Slow systems were slaughtered at Ardenne Volaille SA (Bertrix, Belgium). The chickens were slaughtered under standardized commercial conditions, including electrical stunning, at an approximate line speed of 2000 birds/h. After slaughter, carcasses were chilled using a dynamic air chilling system, transported in a cooling truck (4 °C) to the laboratory at Ghent University, and stored for 24 h at 2 to 4 °C before further processing. Detailed information regarding certain pre-slaughter and processing conditions, including transport duration, lairage time, scalding conditions, chilling duration, and carcass handling procedures, was not available. Therefore, effects of slaughterhouse-specific factors on post-mortem metabolism and meat quality traits cannot be completely ruled out.

On the second day post-slaughter, the skin was removed from the breast and thigh meat cuts, which were then finely ground separately using an electric food grinder (Moulinex DP810; Moulinex, Paris, France). All steps were conducted at 7 °C in a cooling room. Immediately, 40 ± 1 g of ground meat was placed in petri dishes to assess oxidative stability. Grinding and standardisation of the sample size were carried out to obtain more homogeneous samples and to reduce variation caused by differences in the size of the cuts resulting from differences in bird weight. It should be realized that disruption of muscle structure during grinding increases oxygen exposure and promotes interactions between pro-oxidants and oxidation substrates, thereby likely accelerating the onset of oxidation processes (Shimizu and Iwamoto, 2022). Concurrently, aliquots of the ground meat samples were stored at −80°C for later analysis of the fatty acid profile, heme-Fe content and antioxidant enzymes activity.

### Oxidation analyses

#### Color oxidation

The meat in petri dishes was covered with oxygen-permeable polyethylene film (sourced from a local supermarket, Delhaize, with a thickness of 0.010 mm) to limit dehydration and exposed to light (1600 - 2200 lux) for 168 h in the fridge at 2 to 4°C. Although the supplier did not provide specific permeability details for the film, Massey (2002) reports that similar low-density polyethylene films typically have an oxygen permeability ranging from 255 to 470 cm^3^ × mm × m⁻² x 24 h⁻¹ × atm⁻¹ and a water vapor transmission rate of 1.25 to 1.85 g × mm × m⁻² × 24 h⁻¹. Lightness (L*), redness (a*), and yellowness (b*) were measured in duplicate after 0, 48, 96 and 168 h of display, using a Hunterlab Miniscan color meter (D65 light source, 10° standard observer, 45°/0° geometry, 1-inch light surface, white standard) to estimate color stability. L* is a measure of lightness in which 0 equals black and 100 equals white, and a* and b* represent the hue dimension with positive values denoting redness and yellowness, respectively. To assess the color stability, the difference (0 – 168 h) in lightness (**Δ*L***), redness (**Δ*a***), yellowness (**Δ*b***) and total color difference Δ*E* (Δ*E* = [(Δ*L*)^2^ + (Δ*a*)^2^ + (Δ*b*)^2^]1/2) was calculated (King et al., 2023). At the end of the display period, the samples were vacuum packed and frozen at −80°C for later analysis of lipid and protein oxidation.

#### Lipid oxidation

Thiobarbituric acid reactive substances (**TBARS**) were measured spectrophotometrically in duplicate on samples collected after 168 h of display, based on Tarladgis et al. (1960). Briefly, 10 g of thawed meat sample was mixed with distilled water (70 mL) and butylated hydroxytoluene (1 mL) and homogenized using an Ultra-Turrax (T25, IKA Janke and Kunkel, Germany) at 13,000 rpm for 30 s. Then, 3 mL of hydrochloric acid (HCl, 4 M) was added prior to distillation. Subsequently, 1 mL of thiobarbituric acid reagent was mixed with 5 mL of the collected distillate in glass tubes and heated in boiling water for 35 min. Afterwards, the tubes were cooled to room temperature under tap water, and the absorbance was measured at 532 nm using a spectrophotometer (Genesys 10S UV–VIS, Thermo Scientific, Madison, USA). A standard curve was prepared from serial dilutions of 1,1,3,3-tetramethoxypropane and analyzed under the same conditions as the samples. The absorbance values were compared to the standard curve to determine malondialdehyde (**MDA**) concentrations, expressed in nmol MDA/mL distillate. These values were converted to mg MDA/kg meat using the following equation:

TBARS (mg MDA / kg meat) = (nmol MDA/mL × 72g/mol × 100 mL) / (10 g meat x 1000)

where 72 g/mol is the molecular weight of MDA, 100 mL is the distillate volume, and 10 g is the initial meat sample weight.

#### Protein oxidation

Protein oxidation was measured in duplicate on samples after 168 h of display by the formation of protein carbonyl compounds (**PCC**), according to a method modified from Ganhão et al*.* (2010). Briefly, in microtubes, three aliquots of homogenized meat (each 200 μL from the 1:10 meat-to-phosphate buffer solution) were combined with ice-cold trichloroacetic acid (TCA). One aliquot was used as a blank, whereas the others were treated with 2,4-dinitrophenylhydrazine (**DNPH**) to form stable 2,4-dinitrophenylhydrazone complexes. Following a covalent reaction, the carbonyl groups of proteins formed a stable 2,4 dinitrophenylhydrazone. The microtubes were then vortexed and incubated for 1 h, at room temperature, on a shaker at 350 rpm, fully covered from light with aluminum foil. Subsequently, 10% ice-cold TCA was added for precipitation and excess DNPH and fat was removed by three washes with ethanol/ethyl acetate (1:1, v/v). Afterwards guanidine HCl (6 M) was added to dissolve proteins. Absorbance was measured spectrophotometrically, firstly at 280 nm and secondly at 370 nm (Genesys 10S UV–VIS, Thermo Scientific, Madison, USA) to quantify the PCC. The PCC concentrations were calculated by measuring the amount of 2,4-DNPH incorporated per mg of protein, using the following formula:

C_hydrazone_ / C_protein_ = A_370_ / (εhydrazone_370_ × (A_280_ - A_370_ × 0.43)) × 1000

where εhydrazone_370_ is the molar extinction coefficient of hydrazones at 370 nm and 0.43 represents the ratio εhydrazone_280_ / εhydrazone_370_ used to correct for hydrazone absorbance at 280 nm. Results were expressed as nmol carbonyls per mg protein.

### Fatty acids analysis

Lipids were extracted in duplicate from both meat and feed samples using chloroform/methanol (2:1, v/v), and fatty acids were analyzed as described by Raes et al. (2001). After addition of NaOH/MeOH followed by HCl/MeOH, fatty acid methyl esters (**FAME**) were analyzed using a gas chromatograph (HP 7890A, Agilent Technologies, Diegem, Belgium), equipped with a fused silica SP-2560 capillary column (75 m × 0.18 mm i.d. × 0.14 µm thickness; Supelco Analytical, Bellefonte, PA, USA) and a flame ionization detector. A volume of 1 µL of the FAME solution was injected into the gas chromatograph. Hydrogen was used as the carrier gas at a constant flow rate of 1 mL/min. Peaks were identified by comparing the retention times corresponding with standards (GLC463, Nu-Chek-Prep, Elysian, MN, USA). Nonadecanoic acid (C19:0) was added as an internal standard for quantification.

The fatty acid profile was reported as the sum of total fatty acids, including saturated fatty acids (**SFA**; capric acid, C10:0; lauric acid, C12:0; myristic acid, C14:0; pentadecanoic acid, C15:0; palmitic acid, C16:0; heptadecanoic acid, C17:0; stearic acid, C18:0; arachidic acid, C20:0; behenic acid, C22:0; lignoceric acid, C24:0), monounsaturated fatty acids (**MUFA**; myristoleic acid, C14:1; palmitoleic acid, C16:1; heptadecenoic acid, C17:1; oleic acid, C18:1 cis-9; vaccenic acid, C18:1 cis-11; eicosenoic acid, C20:1; erucic acid, C22:1; nervonic acid, C24:1), and PUFA. The PUFA fraction included n-3 polyunsaturated fatty acids (**n-3 PUFA**; α-linolenic acid (**ALA**), C18:3n-3; eicosatrienoic acid, C20:3n-3; eicosatetraenoic acid, C20:4n-3; eicosapentaenoic acid, C20:5n-3; docosapentaenoic acid, C22:5n-3; docosahexaenoic acid, C22:6n-3) and n-6 polyunsaturated fatty acids (**n-6 PUFA**; linoleic acid, C18:2n-6; γ-linolenic acid, C18:3n-6; eicosadienoic acid, C20:2n-6; dihomo-γ-linolenic acid, C20:3n-6; arachidonic acid, C20:4n-6; docosatetraenoic acid, C22:4n-6; docosapentaenoic acid, C22:5n-6).

The peroxidability index (**PI**), which reflects the susceptibility of lipids to oxidative degradation, was calculated by assigning weights to fatty acids based on their degree of unsaturation, using the formula of Holman (1954): PI = (MUFA × 0.025) + (C18:2n-6 × 1) + (C18:3n-6 + C18:3n-3) × 2) + (C20:4n-6 + C20:3n-6 + C20:4n-3) × 4) + (C22:4n-6 + C22:5n-6 + C20:5n-3 + C22:5n-3) × 6) + (C22:6n-3 × 8). Long-chain fatty acid biosynthesis indices were calculated using product-to-precursor ratios, which reflect integrated fatty acid desaturase and elongase activity (Boschetti et al., 2016; Poureslami et al., 2010). The n-6 fatty acid conversion index was expressed as C20:4n-6/C18:2n-6, and the n-3 index as (C20:5n-3 + C22:6n-3)/C18:3n-3.

### Heme-Fe

The heme-Fe content was determined following a modified method of Hornsey (1956). In duplicate, 5 g of meat was homogenized with an acetone/H_2_O/HCl (20/1/0.5) extraction medium at 13,000 rpm for 30 s. The homogenate was kept in complete darkness for 1 h at room temperature to convert all heme pigments. The supernatant was filtered through Schleicher & Schuell filter paper (grade 5893, diameter 110 mm), and the absorbance was measured at 640 nm using a Genesys 10S UV-Vis spectrophotometer. Heme-Fe was calculated using the formula:

heme-Fe = (hematin concentration × atomic weight of iron) / molecular weight of hematin.

### Antioxidant enzyme activities

The analysis of antioxidant enzymes GSH-Px and SOD began with frozen samples. An ice-cold phosphate buffer solution at pH 7 was used as the extraction medium. Two g of ground meat was mixed with 8 mL ice cold buffer, homogenized by ultraturrax (T25, IKA Janke and Kunkel, Germany), and centrifuged (10000 G, 15 min., 4°C) to prepare meat extracts. The supernatants were filtered through glass wool, and the extracts were kept on ice at all times for the measurement of GSH-Px and SOD activities.

#### Glutathione peroxidase

The GSH-Px activity was determined by measuring the oxidation of NADPH according to Hernández et al. (2004). 30 μL of meat extract, 200 μL of medium solution, 10 μL of NADPH solution and 10 μL of H_2_O_2_ solution were added to a 96-well plate. The temperature was set at 25°C and the absorbance was measured with a Tecan absorbance reader (Type Infinite M Nano) at 340 nm during 300 s. One unit of GSH-Px activity was defined as the amount of enzyme required to oxidize 1 µmol of NADPH per g of meat in one minute at 25°C.

#### Superoxide dismutase

The SOD activity assay was performed using a modified method described by Marklund and Marklund (1974) by measuring the inhibition of pyrogallol autoxidation. Prepared meat extract was used in six different volumes (0, 2, 4, 6, 8 and 10 μL) with volumes of distilled water (17, 15, 13, 11, 9, and 7 μL, respectively) added to maintain the same total volume. To each well, 275 μL of Tris-cacodylic buffer (pH 8.2, 50 mM with 1 mM DTPA) and 8 μL of pyrogallol solution (15 mM) were added. Absorbance was monitored using Tecan absorbance reader (Type Infinite M Nano) at 420 nm during 600 s at 25°C. One unit of enzyme activity was defined as the amount of sample needed to inhibit the reaction by 50% at 25°C and pH 8.2.

### Statistical analysis

Data analysis was conducted using R (version 4.3.1) and RStudio (version 2023.09.0). Data were analyzed separately for breast and thigh meat. Normality assumptions were evaluated through visual inspection of model residuals (Q-Q plots and histograms) and formally verified using Shapiro-Wilk tests (P ≥ 0.05). Linear mixed-effects models were fitted using the lme4 package. L*, a* and b* values were analyzed with production system, display time, and their interaction as fixed effects, with sample nested within farm included as a random effect to account for the repeated measurements obtained from the same sample during the display period. Color stability parameters (Δ*L*, Δ*a*, Δ*b* and Δ*E*) were analyzed with production system as a fixed effect and farm ID as a random effect. TBARS, PCC, fatty acid profile, heme-Fe, and antioxidant enzyme activities were analyzed with models including production system as a fixed effect, storage time (the duration between slaughter and laboratory analysis) as a fixed covariate, and farm ID as a random effect. Estimated marginal means for all models described above were obtained using the emmeans package and compared using Tukey-adjusted pairwise comparisons. The significance level was set at P < 0.05.

Principal Component Analysis (**PCA**) was performed using the factoextra and ggplot2 packages in R as an exploratory multivariate technique to visualize relationships among variables and production systems by reducing the dimensionality of the dataset into principal components (**PC**). Separate PCA analyses were conducted for breast and thigh meat using individual animal observations. Variables included color measurements (L*, a*, and b* at 0, 48, 96, and 168 h of display), color stability parameters, TBARS, PCC, fatty acid composition, heme-Fe content, and antioxidant enzyme activities. Color measurements obtained at different display times were included directly in the PCA and were not summarized prior to analysis. All variables were standardized (mean-centered and scaled by standard deviation) prior to analysis to ensure comparability among variables measured on different scales. The first two principal components (PC1 and PC2), which explained the largest proportion of the total variance, were used for visualization, and the variance explained by each component was expressed as a percentage of the total variance. PCA was used solely as an exploratory visualization tool and not for statistical inference.

## Results

### Color, lipid and protein oxidation

The L*, a*, and b* values of breast and thigh meat were influenced by production system throughout the display period (P < 0.001), as shown in Table 2. In breast meat, the L* values were higher for Ind_Fast and Ind_Fastmed compared to Out_Med and Out_Slow after 0, 48 and 168 h of display, whereas after 48 h, Ind_Fastmed had higher L* values than the other groups. For thigh meat, the L* values were higher for Ind_Fast and Ind_Fastmed compared to Out_Slow and Out_Med after 0 and 168 h of display, whereas after 48 and 96 h, Ind_Fast had higher L* values compared to Out_Slow and values were intermediate in Ind_Fastmed and Out_Med. The a* and b* values were lower in Ind_Fast and Ind_Fastmed compared to Out_Med and Out_Slow at all time points in breast as well as in thigh meat.

**Table 2:** Color values of breast and thigh meat from different chicken production systems.

		Production system				P Value		
	Display time (h)	Ind_ Fast	Ind_ Fastmed	Out_ Med	Out_Slow	PS	T	PS × T
*Breast meat*								
Lightness (L*)	0	64.35±0.97^a^	64.29±0.98^a^	58.56±0.95^b^	60.43±0.96^b^	<0.001	<0.001	0.013
	24	62.12±1.08^b^	63.82±1.03^a^	56.66±0.91^c^	60.55±0.96^b^			
	96	61.63±1.00^a^	61.72±1.00^a^	55.45±0.93^c^	58.83±0.95^b^			
	168	61.30±0.97^a^	61.11±0.98^a^	53.64±0.89^c^	57.35±0.94^b^			
Redness (a*)	0	5.46±0.37^b^	5.40±0.37^b^	10.23±0.36^a^	8.79±0.37^a^	<0.001	<0.001	<0.001
	24	6.27±0.39^c^	5.93±0.40^c^	10.00±0.36^a^	8.14±0.37^b^			
	96	6.03±0.39^c^	5.50±0.38^c^	9.88±0.36^a^	7.73±0.36^b^			
	168	5.65±0.37^c^	5.03±0.37^c^	9.79±0.36^a^	7.83±0.36^b^			
Yellowness (b*)	0	18.66±0.78^b^	18.78±0.80^b^	25.17±0.76^a^	24.11±0.77^a^	<0.001	<0.001	<0.001
	24	18.35±0.81^b^	18.48±0.81^b^	23.56±0.78^a^	22.18±0.78^a^			
	96	18.12±0.81^b^	17.77±0.80^b^	23.59±0.78^a^	22.49±0.77^a^			
	168	18.23±0.78^b^	17.55±0.79^b^	24.31±0.81^a^	23.58±0.80^a^			
*Thigh meat*								
Lightness (L*)	0	64.61±0.99ᵃ	63.41±1.01ᵃ	61.23±0.97ᵇ	57.68±0.98^c^	<0.001	<0.001	<0.001
	24	64.44±1.02ᵃ	62.30±1.05^ab^	62.34±0.97ᵃᵇ	59.63±0.98ᵇ			
	96	63.57±1.02ᵃ	61.78±1.02^ab^	61.21±0.97ᵃᵇ	58.34±0.97ᵇ			
	168	63.04±0.99^a^	62.14±1.01^a^	59.45±0.97^b^	57.50±0.97^c^			
Redness (a*)	0	6.44±0.43^b^	7.61±0.44^b^	10.47±0.42^a^	10.65±0.44^a^	<0.001	<0.001	<0.001
	24	8.22±0.47^b^	8.43±0.49^b^	10.50±0.42ᵇ	11.20±0.44^a^			
	96	7.85±0.47^b^	8.42±0.46^b^	10.85±0.42^a^	10.94±0.42^a^			
	168	7.61±0.43^b^	7.55±0.44^b^	10.48±0.42^a^	10.02±0.42^a^			
Yellowness (b*)	0	19.39±0.70^b^	19.27±0.71^b^	24.12±0.69^a^	22.95±0.73^a^	<0.001	<0.001	<0.001
	24	19.10±0.79^b^	19.12±0.80^b^	23.01±0.69^a^	22.37±0.73^a^			
	96	15.95±0.79^b^	18.84±0.75^b^	23.69±0.69^a^	22.24±0.69^a^			
	168	19.40±0.70^c^	20.15±0.71^c^	24.33±0.69^a^	22.25±0.69^b^			

Abbreviations: Ind_Fast = indoor and fast-growing; Ind_Fastmed = indoor and fast-medium-growing; Out_Med = outdoor and medium-growing; Out_Slow = outdoor and slow-growing. Values are estimated marginal means ± SE (n = 15) obtained from the linear mixed-effects model. Different superscripts ^a,b,c^ within a row indicate significant differences among production systems on a given display time. P values correspond to the overall fixed effects of production system (PS), display time (T), and their interaction (PS × T) obtained from the linear mixed-effects model.

**Fig. 2:**
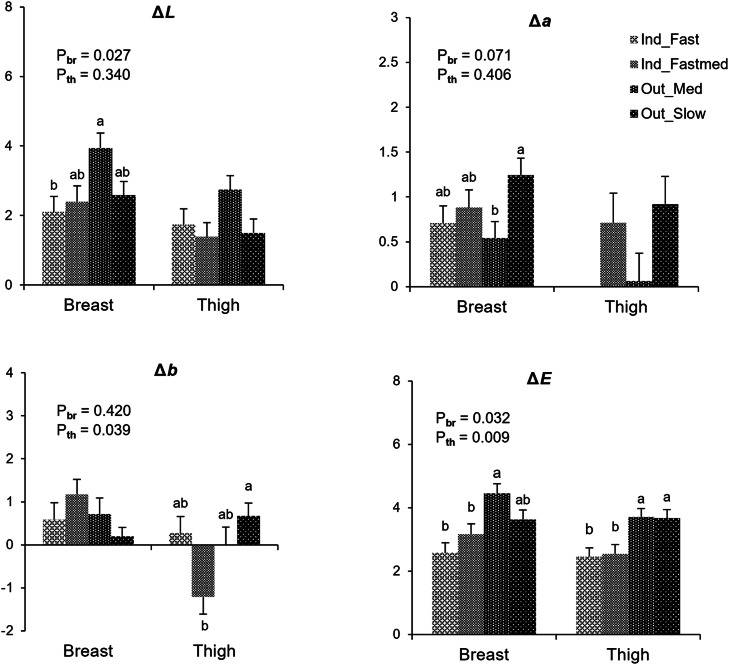
Effects of production system (Ind_Fast, indoor and fast-growing; Ind_Fastmed, indoor and fast-medium-growing; Out_Med, outdoor and medium-growing; Out_Slow, outdoor and slow-growing) on the difference (0 – 168 h) in lightness (Δ*L*), redness (Δ*a*), yellowness (Δ*b*) and total color difference Δ*E* in breast and thigh meat. Bars are estimated marginal means ± SE (n = 15). Different letters at the top of the bars indicate significant differences (P < 0.05). The P values are denoted as P_br_ for breast and P_th_ for thigh.

The differences in color stability are presented in Fig. 2. In breast, the Δ*L* varied significantly among production systems (P = 0.027), with higher values in Out_Med compared to Ind_Fast and intermediate values in Ind_Fastmed and Out_Slow. The Δ*a* was numerically higher in Out_Slow than in Out_Med chickens, with intermediate values in Ind_Fast and Ind_Fastmed chickens; however, this difference was not statistically significant (P = 0.071). The Δ*b* values were not affected (P = 0.420). The Δ*E* was higher in Out_Med compared to Ind_Fast and Ind_Fastmed (P = 0.032), and intermediate in Out_Slow chickens. In thigh meat, Δ*L* and Δ*a* were not affected by production system (P > 0.05). The Δ*b* was higher in Out_Slow compared to Ind_Fastmed chickens and intermediate in Ind_Fast and Out_Med chickens (P = 0.039). Notably, a negative Δ*b* value was observed in thigh meat of Ind_Fastmed chickens. The Δ*E* was higher in Out_Med and Out_Slow chickens compared to Ind_Fast and Ind_Fastmed chickens (P = 0.009).

**Fig. 3:**
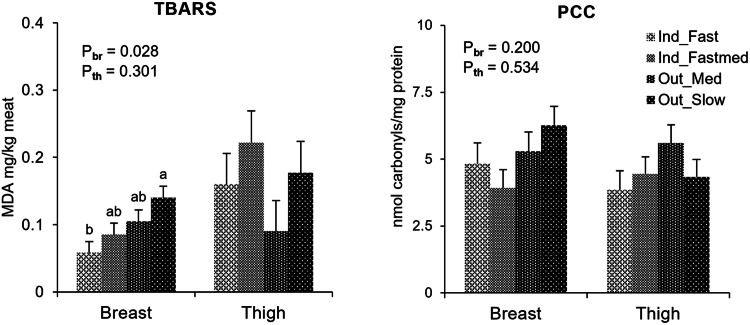
Effects of production system (Ind_Fast, indoor and fast-growing; Ind_Fastmed, indoor and fast-medium-growing; Out_Med, outdoor and medium-growing; Out_Slow, outdoor and slow-growing) on thiobarbituric acid substances (TBARS) and protein carbonyl compounds (PCC) in breast and thigh meat. Bars are estimated marginal means ± SE (n = 15). Different letters at the top of the bars indicate significant differences (P < 0.05). The P values are denoted as P_br_ for breast and P_th_ for thigh.

The TBARS differed among production systems in breast and thigh meat (P < 0.05) (Fig. 3). Higher lipid oxidation in breast meat was observed in Out_Slow chickens than in Ind_Fast (P = 0.028), with intermediate values in Ind_Fastmed and Out_Med. For thigh meat, lipid oxidation was not affected by production system (P = 0.301). No effect of production system was observed on PCC in both meat cuts (P > 0.05) (Fig. 3).

### Fatty acids

The fatty acid profile is presented in Table 3. In both breast and thigh meat, production system did not affect total fatty acid content, SFA, or MUFA (P > 0.05). However, production system affected total PUFA, n-6 PUFA, and n-3 PUFA contents as well as PI (P < 0.05). In both meat cuts, Out_Slow chickens had higher total PUFA and n-6 PUFA contents than chickens of the other production systems, whereas n-3 PUFA contents were higher in Out_Slow and Ind_Fastmed chickens than in Ind_Fast and Out_Med chickens. In breast meat, PI was lowest in Ind_Fast and Ind_Fastmed chickens and highest in Out_Slow chickens, whereas Out_Med chickens showed intermediate values (P = 0.003). In thigh meat, PI was higher in Out_Slow chickens than in the other production systems (P < 0.001).

**Table 3:** Fatty acid composition of breast and thigh meat in chickens from different production systems.

	Production system						
	Ind_ Fast	Ind_ Fastmed	Out_ Med	Out_ Slow	RMSE^1^		P Value
*Breast meat*							
Total FA (g/100 g meat)	0.98	0.85	0.79	0.68	0.30		0.76
*FA (g/100g FAME)*							
SFA	35.21	33.83	34.00	33.00	2.54		0.187
MUFA	32.89	32.02	33.85	31.05	3.26		0.74
PUFA	23.49^b^	25.39^b^	24.52^b^	30.27^a^	2.52		<0.001
n-3-PUFA	2.24^b^	3.16^a^	2.61^b^	3.40^a^	0.48		0.03
*ALA*	1.10^b^	1.75^a^	0.87^b^	1.73^a^	0.28		0.002
*EPA*	0.18	0.25	0.31	0.26	0.08		0.195
*DPA*	0.45	0.81	0.60	0.65	0.15		0.104
*DHA*	0.24	0.32	0.44	0.44	0.16		0.079
n-6-PUFA	21.24^b^	22.22^b^	21.56^b^	26.87^a^	2.26		<0.001
*LA*	16.99^b^	18.63^b^	16.18^c^	21.46^a^	2.04		<0.001
*AA*	2.51	2.53	3.38	3.60	0.69		0.085
PI	40.37^b^	43.68^b^	48.60^ab^	56.04^a^	6.36		0.003
*AA/LA*	0.15^b^	0.14^b^	0.21^a^	0.17^ab^	0.03		0.06
*(EPA+DHA)/ALA*	0.39^b^	0.37^b^	0.82^a^	0.43^b^	0.18		0.002
n-6:n-3	10.02	6.98	7.85	8.02	0.92		0.097
*Thigh meat*							
Total FA (g/100 g meat)	5.77	4.09	3.83	5.40		1.94	0.100
*FA (g/100g FAME)*							
SFA	33.46^a^	35.12^a^	31.96^a^	28.28^b^		2.47	0.001
MUFA	38.57	37.32	43.23	36.50		2.82	0.11
PUFA	22.92^b^	25.16^b^	22.29^b^	30.28^a^		3.13	<0.001
n-3-PUFA	1.87^b^	3.35^a^	2.05^b^	2.87^a^		0.24	<0.001
*ALA*	1.47^b^	2.34^a^	1.63^b^	2.49^a^		0.21	<0.001
*EPA*	0.07	0.08	0.07	0.07		0.01	0.622
*DPA*	0.19	0.29	0.18	0.26		0.06	0.058
*DHA*	0.08^b^	0.11^b^	0.10^b^	0.16^a^		0.05	0.007
n-6-PUFA	21.05^b^	22.26^b^	20.23^b^	27.16^a^		2.92	<0.001
*LA*	19.15^b^	20.34^b^	18.35^b^	24.82^a^		2.66	<0.001
*AA*	1.17	1.20	1.20	1.48		0.35	0.112
PI	32.24^b^	36.27^b^	32.29^b^	43.17^a^		4.74	<0.001
*AA/LA*	0.06	0.05	0.06	0.05		0.01	0.782
*(EPA+DHA)/ALA*	0.10	0.08	0.11	0.09		0.02	0.267
n-6:n-3	11.21^a^	8.82^b^	11.26^a^	8.57^b^		1.16	0.002

Abbreviations: Ind_Fast = indoor and fast-growing; Ind_Fastmed = indoor and fast-medium-growing; Out_Med = outdoor and medium-growing; Out_Slow = outdoor and slow-growing; FA = fatty acids; FAME = fatty acid methyl esters; SFA = saturated fatty acid; MUFA = monounsaturated fatty acid; PUFA = polyunsaturated fatty acid (n-3-PUFA + n-6-PUFA; ALA = α-linolenic acid (C18:3n−3); EPA = eicosapentaenoic acid (C20:5n−3); DHA = docosahexaenoic acid (C22:6n−3); n-3-PUFA = omega-3 polyunsaturated fatty acid (C18:3n-3, C20:3n-3, C20:4n-3, C20:5n−3, C22:5n−3, C22:6n−3); n-6-PUFA = omega-6 polyunsaturated fatty acid (C18:2n−6, C18:3n−6, C20:2n− 6, C20:3n−6, C20:4n−6, C22:4n−6, C22:5n−6); LA = linoleic acid (C18:2n−6); AA = arachidonic acid (C20:4n-6); PI = peroxidizability index. Values are estimated marginal means (n = 15) obtained from the linear mixed-effects model. Different superscripts ^a,b,c^ within a row indicate significant differences among production systems. ^1^RMSE = root mean squares error.

### Heme-Fe

**Fig. 4:**
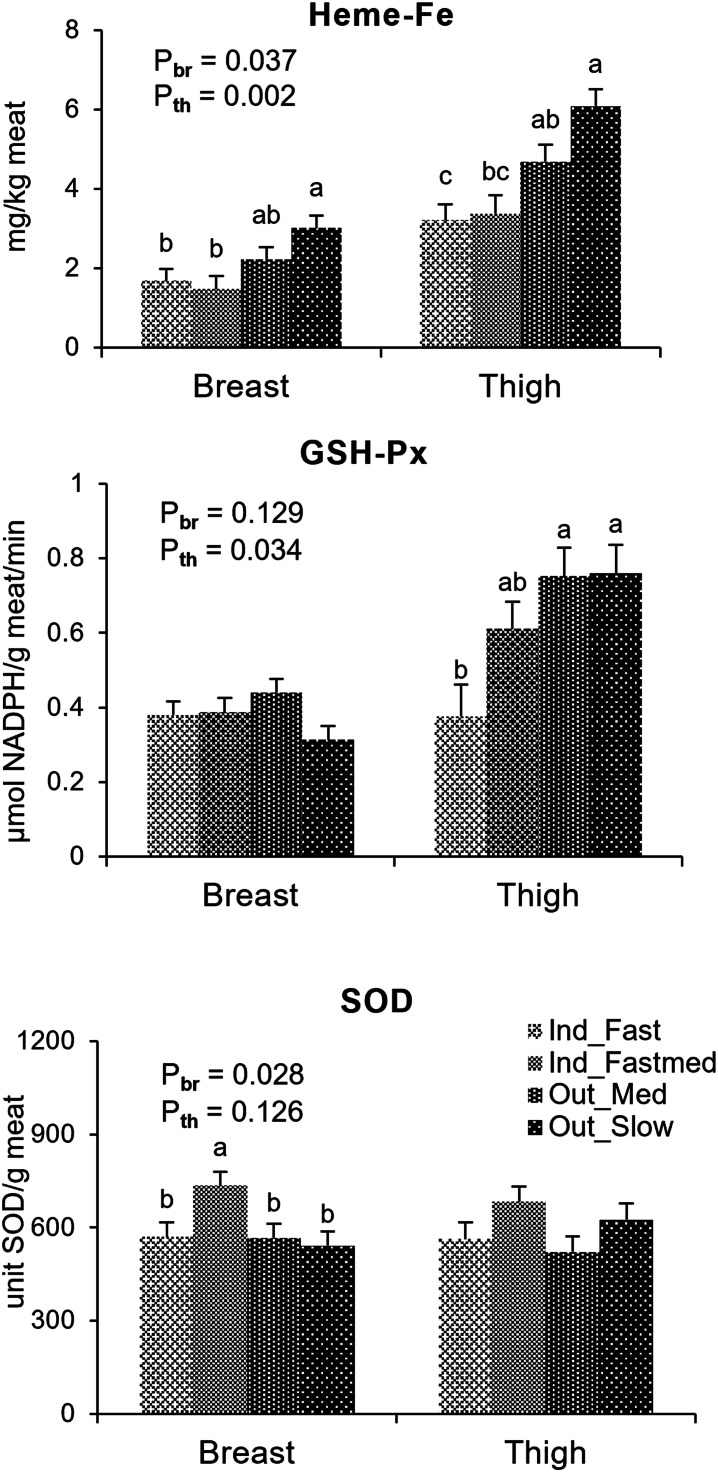
Effects of production system (Ind_Fast, indoor and fast-growing; Ind_Fastmed, indoor and fast-medium-growing; Out_Med, outdoor and medium-growing; Out_Slow, outdoor and slow-growing) on Heme-Fe, and GSH-Px and SOD activity in breast and thigh meat. Bars are estimated marginal means ± SE (n = 15). Different letters at the top of the bars indicate significant differences (P < 0.05). The P values are denoted as P_br_ for breast and P_th_ for thigh.

The heme-Fe content differed among systems in both breast and thigh meat (P < 0.05) (Fig. 4). Breast meat from the Out_Slow system had a significantly higher concentration compared to Ind_Fast and Ind_Fastmed, with an intermediate concentration in Out_Med chickens (P = 0.037). Similarly, in thigh meat, Out_Slow chickens showed higher heme-Fe concentrations than Ind_Fast and Ind_Fastmed, whereas Out_Med chickens also had higher concentrations than Ind_Fast (P = 0.002).

### Antioxidant enzyme activities

The activities of GSH-Px and SOD enzymes are presented in Fig. 4. For breast meat, GSH-Px activity did not differ significantly among production systems (P > 0.05). The SOD activity was higher in Ind_Fastmed compared to the other groups (P = 0.028). In thigh meat, GSH-Px activity was higher in Out_Med and Out_Slow compared to Ind_Fast (P = 0.034) and intermediate in Ind_Fastmed. SOD activity was not affected by production system (P > 0.05).

### Principal component analysis

**Fig. 5:**
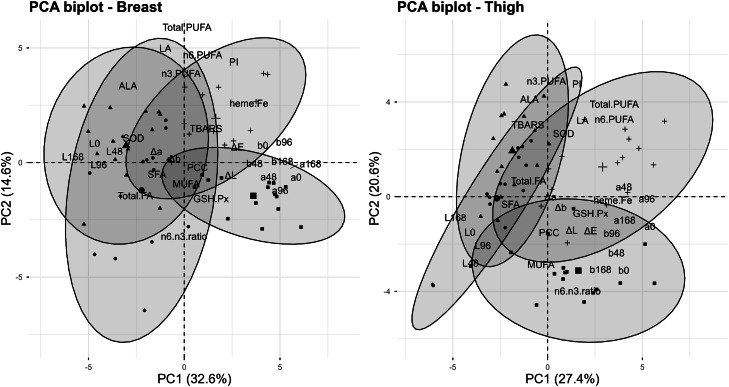
Principal component analysis (PCA) biplots showing the distribution of breast and thigh meat samples from different production systems across the first two principal components (PC1 and PC2), based on measured response variables. Production systems are represented as follows: indoor and fast-growing (●), indoor and fast-medium growing (**▲**), outdoor and medium-growing (■), and outdoor and slow-growing (**+**). The ellipses represent the 95% confidence interval for each production system. The prefixes L, a, and b denote lightness, redness, and yellowness, respectively, whereas the suffixes 0, 48, 96, and 168 denote the display time (h).

PCA biplots for breast and thigh meat are shown in Fig. 5. In breast, 47% of the variance was explained by the first two PC (PC1: 32.6%, PC2: 14.6%). Samples from the Ind_Fast and Ind_Fastmed systems largely overlapped in the PCA space, positioned in the direction of higher L* values, SOD activity, and total fatty acids. Samples from the Out_Slow system were positioned further from the other groups along PC1, in the direction of heme-Fe and TBARS, whereas Out_Med samples were more closely associated with a* values and Δ*L*. Some overlap was observed among all systems for MUFA, PCC, and GSH-Px, whereas n-3 and n-6 PUFA were positioned closer to samples from Ind_Fast, Ind_Fastmed, and Out_Slow.

In thigh meat, 48% of the variance was explained by the first two PC (PC1: 27.4%, PC2: 20.6%). Ind_Fast and Ind_Fastmed samples overlapped in the PCA space, oriented toward higher L*, TBARS, and n-3 PUFA. Out_Slow samples tended to occupy a more distinct region of the PCA space, in the direction of heme-Fe, LA, n-6 PUFA, total PUFA, and Δ*a*. However, unlike in breast meat, TBARS were not associated with the Out_Slow direction. The Out_Slow and Out_Med groups showed partial overlap along the direction of Δ*L*, PCC, and GSH-Px, whereas Out_Med samples extended toward n-6:n-3 ratio, MUFA, b* values, and Δ*E*.

## Discussion

In Europe, poultry production is regulated according to EU standards, encompassing systems that range from the conventional high-input systems characterized by indoor housing and fast-growing genotypes to low-input organic systems with mandatory outdoor access and slower-growing genotypes, and ‘in-between’ systems. A prominent example of the latter is the BCC/ECC system, based on a public commitment to higher animal welfare standards. We evaluated the difference in oxidative stability of breast and thigh meat from chickens raised under four distinct production systems in Belgium; exemplary for the diversity in systems across Europe. Since production systems differ in multiple characteristics, the present comparison should be considered as an observational study which does not allow disentangling the effects of the underlying production practices. We measured the oxidative stability following grinding and displaying the meat under continuous exposure to light at low temperature. One may assume that this protocol accelerated oxidation processes compared to retail display of intact meat cuts under intermittent light conditions.

Meat from the outdoor-raised groups was darker than meat from the indoor-raised groups, consistent with previous studies reporting lighter meat color in fast-growing genotypes than in slower-growing genotypes (Huo et al., 2022; Akyüz and Onbaşılar, 2024). Such differences have been associated with variation in muscle metabolic characteristics and pigment content between fast- and slow-growing genotypes (Huo et al., 2022). The higher myoglobin content, estimated from the heme-Fe content, in meat from outdoor-reared chickens is also consistent with a shift toward more oxidative muscle metabolism and a higher proportion of myoglobin-rich fibres due to greater locomotor activity in slow-growing genotypes (Castellini et al., 2002). In contrast, fast-growing genotypes from indoor-reared chickens may exhibit reduced oxidative metabolism and greater lactate accumulation, contributing to paler meat (Fletcher, 2002; Joiner et al., 2014). Furthermore, high stocking densities in indoor housing systems have been reported to influence muscle metabolism and postmortem muscle characteristics, potentially increasing light scattering and contributing to paler meat appearance (Jung et al., 2022).

Meat redness is predominantly determined by the myoglobin content and its chemical state. The higher color redness in outdoor-raised chickens in the present study is thus not surprising and aligns with previous studies (Samuel et al., 2012). Differences in slaughter age may also have contributed to the observed differences in redness (Castellini et al., 2002). Myoglobin content increases with age, e.g., Carvalho et al. (2017a) compared chickens at 8 and 26 weeks and reported an approximately 10-fold and 4-fold higher myoglobin content in breast and thigh meat, respectively, in the older birds. In our study, the myoglobin concentration was approximately 1.6-fold and 2-fold higher in breast and thigh meat, respectively, in Out_Slow chickens compared with Ind_Fast chickens. These two production systems also differed in slaughter age by approximately 4–5 weeks.

Increased yellowness of meat from chickens with outdoor access may reflect differences in the absorption and deposition of dietary carotenoids, as chickens cannot synthesize these pigments and their deposition depends on both diet and genetic predisposition (Fletcher, 2002; Jlali et al., 2012; Küçükyilmaz et al., 2012). Chickens from the Out_Med system in the present study were fed a corn-based diet, and higher meat yellowness has previously been reported in chickens fed corn-based diets (Lyon et al., 2004). Additionally, slow- and medium-growing genotypes such as Hubbard JA757 have been shown to exhibit greater pigment deposition in muscle tissue when provided with outdoor range access, which has been associated with differences in the utilization and deposition of dietary pigments (Bonnefous et al., 2024).

The Δ*E* values, which account for the combined changes in L*, a*, and b*, are an indicator of color stability (Zareian et al., 2019). Although no universal threshold for visually perceptible color differences in meat systems has been established, values in the range of Δ*E* = 2–6 have been proposed as differences that become noticeable to the human eye (Larraín et al., 2008). In our study, the Δ*E* values fell within this range, suggesting that the color changes occurring during the accelerated display period were likely visible to consumers. In meat systems, visible color changes during storage are commonly associated with oxidative processes affecting myoglobin and the accumulation of metmyoglobin, resulting in reduced color stability and consumer acceptability (Carvalho et al., 2017a).

During aerobic storage, meat color deterioration is primarily driven by oxidative changes in myoglobin. The decrease in redness is generally linked to the oxidation of both oxymyoglobin (red; Fe^2+^) and native myoglobin (purple; Fe^2+^) into metmyoglobin (brown; Fe^3+^), which leads to discoloration (McMillin, 2008; Joseph et al., 2012). The greater decrease in redness observed in Out_Slow chickens may be due to their higher myoglobin and heme-Fe levels. In addition, the ongoing lipid oxidation during storage can further accelerate myoglobin oxidation. The reactive lipid-derived products, such as 4-hydroxynonenal, promote the conversion of oxymyoglobin to metmyoglobin through alkylation of amino acid residues (Faustman et al., 2010; Marcinkowska-Lesiak et al., 2016; Wang et al., 2021). Consistent with this relationship, Out_Slow chickens also exhibited higher lipid oxidation in breast meat after display.

An unexpected large increase in thigh yellowness (b*) was observed in Ind_Fastmed chickens during the display period. Although a precise mechanism is unclear, this effect may be associated with the elevated lipid oxidation observed in this group, as lipid oxidation products can interact with muscle pigments and influence color attributes (Zhang et al., 2011). Pigment alterations during cold storage, particularly metmyoglobin formation, have previously been reported to increase b* values in poultry meat (Marcinkowska-Lesiak et al., 2016).

The fatty acid composition of the finishing diets differed substantially among production systems and was broadly reflected in the corresponding meat fatty acid profiles. In particular, the Out_Slow diet had the highest n-3 and n-6 PUFA content, and concomitantly the highest PI, which was consistent with the highest total PUFA content and PI observed in meat from Out_Slow chickens. In contrast, the fatty acid composition of the Out_Med diet was closer to that of the indoor production systems, which may explain why the fatty acid profile of Out_Med meat more closely resembled that of the indoor groups despite the provision of outdoor access. This suggests that, under the conditions of the present study, the formulated diet was likely a major contributor to the observed meat fatty acid profile, whereas outdoor access alone may not have been sufficient to markedly alter the muscle fatty acid composition. This aligns with the low contribution of pasture intake to total dry matter intake in broilers, reported to be approximately 2.5–4.5% (Ponte et al., 2008). Nevertheless, differences in genotype-related metabolism and physiological adaptation may have influenced the partitioning and deposition of dietary fatty acids. Previous studies have suggested that active chickens may utilize PUFA for physiological functions such as immune responses, thermoregulation, and energy metabolism rather than depositing them in muscle tissue (Küçükyilmaz et al., 2012; Zhu et al., 2011; Cartoni Mancinelli et al., 2022). However, the extent to which increased activity influences PUFA deposition may depend on genotype. Slow-growing genotypes are generally considered better adapted to outdoor production systems and may therefore retain a greater capacity for PUFA deposition in muscle tissue despite higher activity levels (Castellini and Dal Bosco, 2017; Dal Bosco et al., 2021; Mattioli et al., 2025). Therefore, differences in meat fatty acid composition likely reflected the combined influence of multiple production system factors, including diet composition, genotype, and outdoor access.

TBARS values were well below the threshold of 1 mg/kg associated with off-flavors (O'Neill et al., 1998). Nonetheless, higher TBARS levels were observed in the breast meat of Out_Slow chickens, aligning with previous findings of greater lipid oxidation in outdoor-reared chickens (Castellini et al., 2002; Dal Bosco et al., 2016). Several factors may explain this higher lipid oxidation. First, increased physical activity in outdoor-reared animals elevates oxygen consumption and ROS production. Slow-growing genotypes develop more oxidative muscle fibers, higher mitochondrial density, and increased cellular respiration, all of which increase susceptibility to lipid oxidation (Branciari et al., 2009; Kikusato and Toyomizu, 2013; Mattioli et al., 2021). In contrast, fast-growing genotypes (e.g., Ross 308), which are less active and retain more glycolytic muscle fibers, exhibit a greater ability to preserve their tissue antioxidant reserves and reduce post-mortem oxidative stress (Dal Bosco et al., 2010; Mattioli et al., 2017; Huo et al., 2022). Secondly, increased slaughter age has been associated with higher lipid oxidation due to greater heme-Fe levels (Castellini et al., 2002; Castromán et al., 2013; Pellattiero et al., 2020). Third, slow-growing chickens exhibited higher PUFA levels and a correspondingly higher PI (Castellini et al., 2002; Dal Bosco et al., 2012; Domínguez et al., 2019). Previous studies have reported differences in fatty acid metabolism and deposition between slow- and fast-growing genotypes (Dal Bosco et al., 2012; Boschetti et al., 2016). Together, these factors may have contributed to the greater susceptibility of breast meat from Out_Slow chickens to lipid oxidation during storage.

Lipid oxidation did not differ among production systems in thigh meat, although average values and numerical differences among systems were larger compared to breast meat. This contrasts with previous studies that reported differences in thigh meat lipid oxidation among genotypes and production systems, associated with variation in antioxidant capacity, muscle metabolism, and adaptation to different rearing conditions (Fotou et al., 2024; Mattioli et al., 2017, 2025). However, it should be realized that outdoor access was substantially reduced on several farms due to avian influenza restrictions, which limited the opportunities for grazing and physical activity in the outdoor production systems. As a result, potential effects of locomotor activity and forage intake may have been less pronounced than expected.

The response of antioxidant enzymes to production system differed between breast and thigh muscles. In the present study, production system differences in GSH-Px activity were observed only in thigh meat, whereas SOD activity differed among production systems only in breast meat. These findings suggest that antioxidant enzyme responses to production system characteristics were not uniform across muscle types. Previous studies have similarly reported differences in antioxidant enzyme activities between breast and thigh muscles, reflecting their distinct fiber-type composition, oxidative metabolism and antioxidant requirements (Bekhit et al., 2013; Korzeniowska et al., 2024). Furthermore, Coudert et al. (2023) demonstrated that the contribution of antioxidant enzymes to oxidative defense may vary according to genotype and age. However, despite the observed differences in enzyme activities, corresponding differences in lipid oxidation were not consistently detected across muscles and production systems, indicating that the relationship between antioxidant enzyme activity and oxidative stability is likely influenced by multiple interacting factors.

In contrast to the differences observed in several indicators related to lipid oxidation and antioxidant status, PCC levels were not affected by production system. PCC levels in breast and thigh meat reached ∼5 nmol/mg by 168 h (∼ day 7) post-slaughter in our study, exceeding values reported by Carvalho et al. (2017b), who observed an increase from ∼0.25 nmol/mg at day 0 to ∼3 nmol/mg by day 7. Protein oxidation is generally considered a more complex process than lipid oxidation because it depends on the interaction of multiple pro-oxidant and antioxidant factors acting simultaneously within the muscle matrix (Estévez and Heinonen, 2010). Therefore, although production system differences were observed for heme-Fe content, fatty acid composition, and antioxidant enzyme activities, these differences were not reflected in PCC levels. This finding suggests that the variation in these factors may not have been sufficient, individually or collectively, to result in measurable differences in protein oxidation under the conditions evaluated. Similar observations were reported by Castromán et al. (2013), who found no significant differences in protein oxidation between organic and conventional production systems in either breast or thigh meat. This indicates that protein oxidation may be less sensitive to production system differences than indicators of lipid oxidation and color stability.

The findings of the present study should be interpreted within the context of an observational comparison of commercial production systems. Consequently, the differences observed in meat oxidative stability and related compositional traits reflect the combined influence of multiple production system characteristics, including genotype, diet composition, housing conditions, slaughter age, management practices, and outdoor access, rather than the effect of any single factor. Furthermore, a limitation of the present study is the relatively low power with 5 farms per production system and 3 broilers per farm sampled for analyses. Although the production system typologies in the present study were clearly differentiated, we cannot guarantee that the farms were fully representative of the large variation that is typically encountered in commercial practice, even in systems under label standards. Nevertheless, the findings suggest that differences in oxidative stability may occur among meats from production systems. Future studies involving larger datasets and more controlled experimental designs are warranted to clarify the relative contribution of specific production system factors to chicken meat oxidative stability.

## Conclusions

This study revealed that differences in chicken production system likely influenced the pro-oxidant and antioxidant profile in the meat, thereby affecting its susceptibility to oxidative reactions in distinct ways. Significant meat color differences were observed, with meat from indoor-raised chickens with fast-growing genotypes being paler, less red, and less yellow, whereas outdoor-raised, medium- and slow-growing chickens showed darker meat with slightly lower color stability over storage. Despite differences in fatty acid profile and heme-Fe content and display model conditions that stimulated oxidation compared to retail display of intact meat cuts, only modest differences in lipid oxidation were observed after 7 days of display, and TBARS values across all systems remained below the threshold for rancidity. Notably, protein oxidation was relatively uniform across systems. Given the growing consumer interest in less intensive and welfare-friendly chicken production systems, future studies should explore strategies to optimize oxidative stability without compromising the benefits of alternative chicken production systems.

## Ethics approval

Not applicable.

## CRediT authorship contribution statement

**Zeshan Ali:** Writing – original draft, Methodology, Data curation, Conceptualization. **Massimiliano Petracci:** Writing – review & editing, Conceptualization. **Cécile Berri:** Writing – review & editing, Funding acquisition, Conceptualization. **Els Vossen:** Writing – review & editing, Methodology. **Eline Kowalski:** Supervision. **Stefaan De Smet:** Writing – review & editing, Supervision, Resources, Project administration, Funding acquisition, Conceptualization.

## Disclosures

The authors declare that they have no known competing financial or personal interests that could have influenced the work reported in this paper.
